# 胸腔膈胸膜源性罕见巨大孤立性纤维瘤1例

**DOI:** 10.3779/j.issn.1009-3419.2012.01.13

**Published:** 2012-01-20

**Authors:** 远大 程, 春芳 张, 阳 高, 硕 董

**Affiliations:** 410008 长沙，中南大学湘雅医院心胸外科 Department of Cardiothoracic Surgery, Xiangya Hospital, Central South University, Changsha 410008, China

## 1 临床资料

患者，男，35岁，因咳嗽1年、气促5个月入院。既往体健。查体：生命体征平稳，气管右移，左侧胸廓饱满，胸骨无压痛，左肺叩诊呈实音，右肺叩诊清音，左肺呼吸音极低，右肺呼吸音清，双肺未闻及明显干湿性啰音。心尖搏动于右侧第5肋间胸骨旁1 cm，心律整齐无杂音。CT示（图 1）：左上、下支气管狭窄，左上下舌段支气管闭塞，远端肺组织不张，左侧胸腔呈一较均匀一致的高密度影，平扫CT值约36 Hu，增强后为35 Hu-58 Hu。完善术前检查后于2011年9月7日在全麻下经左侧第5肋后外侧切口进胸，术中见肿瘤充满左侧胸腔，包膜完整，血运丰富，与肺叶和心包稍有粘连，无法探及瘤蒂位置，无胸腔积液。手术采用分块切除法，至肿瘤完整切除，肿瘤重约7 kg，剖面呈灰白色，瘤蒂位于左膈面，基底部较宽。病检结果示：少量梭形细胞成分，倾向孤立性纤维性肿瘤。免疫组化示：Bcl-2（+）、CD34（+）、CD117（-）、CR（-）、MC（-）、S-100（-）（图 2）。术后复查胸片示肺复张良好（图 3），术后13天痊愈出院。

**1:**
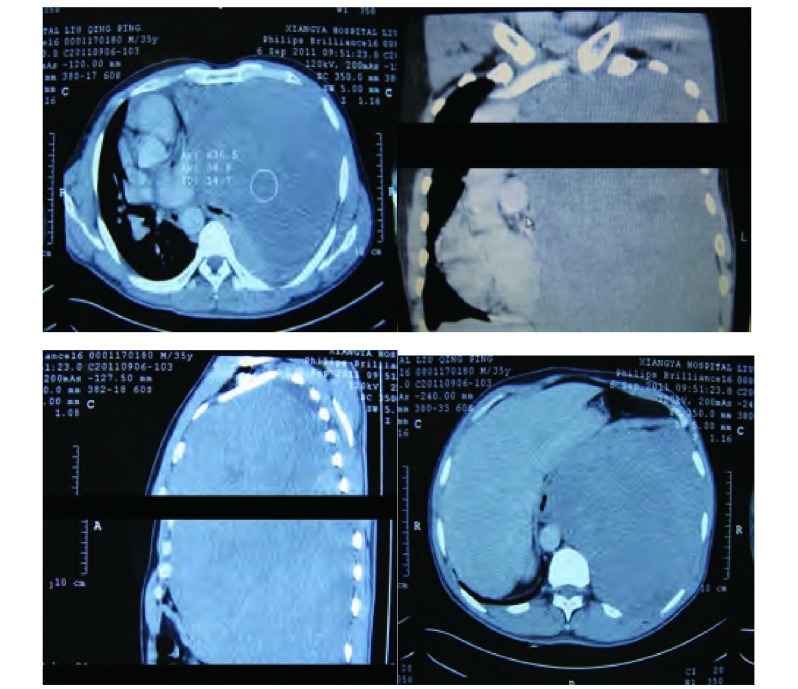
术前CT示肿块填满左侧胸腔，下极达肝脏平面，左肺被完全压缩。 Preoperative CT shows that the mass fills the left thoracic cavity with the low pole in the plane of the liver and the left lung is compressed completely.

**2:**
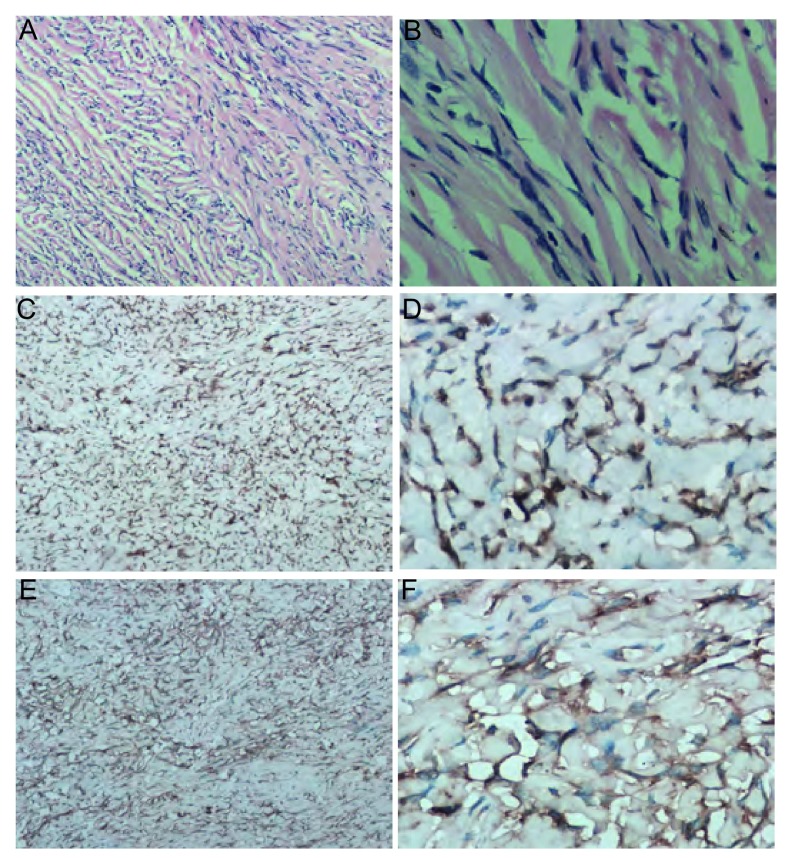
A、B为SFTP的HE染色病理图片，可见梭形细胞成分，多细胞和少细胞区交替存在，部分区域可见扩大的分支血管（A：HE, ×100; B：HE, ×400）。C、D、E、F为SFTP的免疫组化病理图片。C、D示Bcl-2阳性图片，可见细胞膜被染成棕黄色；E、F示CD34阳性图片，可见细胞质被染成棕黄色（C：Bcl-2阳性，×100；D：Bcl-2，×400；E：CD34阳性，×100；F：CD34阳性，×400）。 A and B are the SFTP HE staning pathological pictures, which show alternation of multi-cells and hypo-cells region and some enlarged branch vessels, besides of fusiform cells compnent (A: HE, ×100; B: HE, ×400); C, D, E and F are the SFTP immunohistochemical pathology images. C and D are the pictures of the Bcl-2 positive which show membranous staning; E and F are the pictures of CD34 positive which show cytoplasm staning (C: Bcl-2 positive, ×100; D: Bcl-2 positive, ×400; E: CD34 positive, ×100; F: CD34 positive, ×400).

**3:**
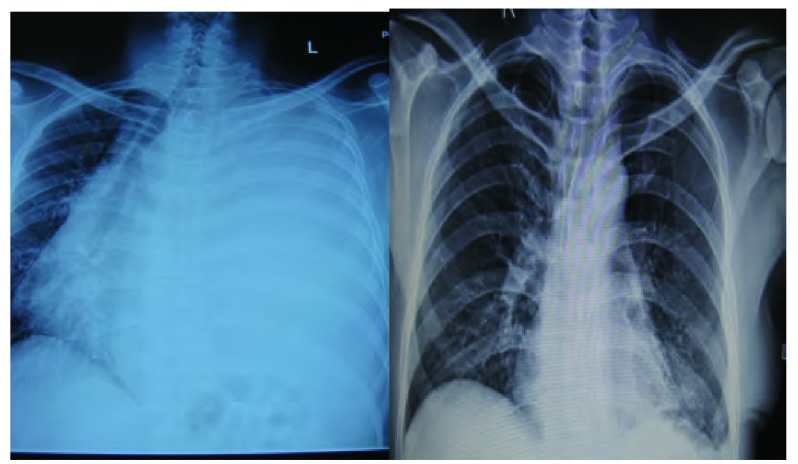
术前胸片，可见纵膈明显向右移位，左侧肺野高密度影。术后复查胸片示肺复张良好，左下可见引流管影。 Chest radiography before surgery shows mediastinum shift to right apparently and high density image in the whole left lung field. However, the left lung-re-expansion well and drainage tube seen in the postoperative chest radiography.

## 2 讨论

胸膜孤立性纤维瘤（solitary fibrous tumor of pleura, SFTP）比较少见，仅占胸膜源性肿瘤的5%^[1]^。自1931年Klemperer和Rabin报告了第1例SFTP以来，国内外有关胸腔内孤立性纤维瘤（solitary fibrous tumor, SFT）个案的报道仅有800多例^[2]^。Cardillo^[1]^统计了55例SFTP结果发现，48例患者肿瘤来自脏层胸膜（87%），7例来自壁层胸膜（13%）。SFTP的发病年龄多在50岁-70岁，男女发病率相当^[3]^。SFTP直径多在8 cm-14 cm^[4]^，＞20 cm的SFT比较少见，＞30 cm则非常罕见。2011年Trivino和Furukawa^[5, 6]^各自报道了1例SFTP，Trivino报道的是1例60岁女性患者，肿瘤来自右中肺叶脏层胸膜，大小约为30 cm×18 cm×20 cm。Furukawa报道的是1例57岁男性患者，肿瘤大小约20 cm×19 cm×15 cm，经两次手术完整切除肿瘤。本例特点为：中年男性患者，肿瘤来源于左膈胸膜，肿瘤体积巨大，大小约28 cm×18 cm×18 cm，在临床上非常罕见。

SFTP的诊断依靠免疫组化，多表达为Bcl-2（+）、CD34（+）^[7]^。SFTP可表现为良性和恶性生长两种方式。良性的SFTP多来自脏层胸膜，有蒂，直径一般＜10 cm，镜下细胞稀疏，分裂较少^[2]^。此病例肿瘤来自壁层胸膜，留蒂面积较大，肿块巨大，不排除恶性可能，术后应定期复查。有研究^[2]^认为恶性SFTP术后有63%的复发率。目前术后1个月复查胸片，未见明显异常。

SFTP的治疗以手术完整切除为主要治疗方式^[8]^。对于巨大SFTP的手术治疗，临床上常遇到以下几种困难：①肿瘤较大，无法直接判断瘤蒂的位置。此病例瘤蒂位于膈面，术中因肿瘤体积较大无法探及，术前CT检查未见明显瘤蒂者可通过多普勒彩超、血管造影等判断瘤内血运及瘤蒂的位置；②肿瘤血运丰富，术中易出血。此病例肿瘤实质血运较差而包膜血运丰富，手术予以分块切除，如遇到实质血运丰富的肿瘤，术中出血很难控制，术前需充分备血；③切口选择问题。目前，手术切口一般选择第5肋骨后外侧切口，有报道^[9]^认为第5、6两肋骨行后外侧切口，无论哪种切口均需一定长度，创伤较大；④术中如何判断肺受压损伤的程度以及是否需要一并行肺叶切除或全肺切除的问题目前尚无统一认识。此例患者CT示左肺明显受压不张，术中切除肿瘤后左肺自行缓慢膨起，未见明显肺组织坏死，未予任何处理，术后复查胸片恢复良好；⑤术后复张性肺水肿问题等。能够恰当的处理和解决以上问题，则胸腔巨大SFT的手术成功率将会提高，患者术后的并发症发生率将减少。

## References

[1] Cardillo G, Facciolo F, Cavazzana AO (2000). Localized (solitary) fibrous tumors of the pleura: an analysis of 55 patients. Ann Thorac Surg.

[2] Robinson LA (2006). Solitary fibrous tumor of the pleura. Cancer Control.

[3] Thorgeirsson T, Isaksson HJ, Hardardottir H (2010). Solitary fibrous tumors of the pleura: an estimation of population incidence. Chest.

[4] Regal MA, Al Rubaish AM, Al Ghoneimy YF (2008). Solitary benign fibrous tumors of the pleura. Asian Cardiovasc Thorac Ann.

[5] Trivino A, Cozar F, Congregado M (2011). Giant solitary fibrous tumor of the pleura. Interact Cardiovasc Thorac Surg.

[6] Furukawa N, Hansky B, Niedermeyer J (2011). A silent gigantic solitary fibrous tumor of the pleura: case report. J Cardiothorac Surg.

[7] Gold JS, Antonescu CR, Hajdu C (2002). Clinicopathologic correlates of solitary fibrous tumors. Cancer.

[8] Bini A, Brandolini J, Davoli F (2009). Solitary fibrous tumor of the pleura: surgery and clinical course in 18 cases. Asian Cardiovasc Thorac Ann.

[9] Fu X, Zhao H, Sun GL (2011). Giant solitary fibrous tumor of the pleura complicated pleural effusion: case report. Jinlin Medi J.

